# Genome sequencing identifies a large non-coding region deletion of *SNX10* causing autosomal recessive osteopetrosis

**DOI:** 10.1038/s10038-022-01104-2

**Published:** 2022-12-16

**Authors:** Prajna Udupa, Debasish Kumar Ghosh, Neethukrishna Kausthubham, Hitesh Shah, Sandip Bartakke, Ashwin Dalal, Katta M Girisha, Gandham SriLakshmi Bhavani

**Affiliations:** 1 Department of Medical Genetics, Kasturba Medical College, Manipal, Manipal Academy of Higher Education, Manipal, India; 2 Department of Pediatric Orthopedics, Kasturba Medical College, Manipal, Manipal Academy of Higher Education, Manipal, India; 3 Department of Clinical Hematology, Aditya Birla Memorial Hospital, Pune, India; 4 Diagnostics Division, Centre for DNA Fingerprinting and Diagnostics, Hyderabad, India

**Keywords:** Genetics research, Rare variants

## Abstract

Autosomal recessive osteopetrosis (ARO) is a rare genetic disorder caused by impaired osteoclast activity. In this study, we describe a 4-year-old boy with increased bone density due to osteopetrosis, autosomal recessive 8. Using genome sequencing, we identified a large deletion in the 5′-untranslated region (UTR) of *SNX10* (sorting nexin 10), where the regulatory region of this gene is located. This large deletion resulted in the absence of the *SNX10* transcript and led to abnormal osteoclast activity. *SNX10* is one of the nine genes known to cause ARO, shown to interact with V-ATPase (vacuolar type H( + )-ATPase), as it plays an important role in bone resorption. Our study highlights the importance of regulatory regions in the 5′-UTR of *SNX10* for its expression while also demonstrating the importance of genome sequencing for detecting large deletion of the regulatory region of *SNX10*.

## Introduction

Osteopetrosis is a form of skeletal dysplasia characterized by increased bone density due to decreased osteoclast activity [1]. The severity of this disease ranges from asymptomatic to very mild to fatal in infancy. Autosomal recessive osteopetrosis (ARO) is a rare, heterogeneous group of osteopetrosis disorders with an estimated incidence of 1 in 250,000 newborns [2]. The characteristic features of this disease are increased bone density, visual impairment, hepatomegaly, splenomegaly, and bone marrow failure. *SNX10* is one of nine genes (*OSTM1, CLCN7, CA2, TNFSF11, TNFRSF11A, PLEKHM1, TCIRG1*, and *FERMT3*) responsible for autosomal recessive osteopetrosis [3]. SNX10 has been shown to interact with V-ATPase (vacuolar type H( + )-ATPase), which helps in pumping protons into the osteoclast bone lacunae [4]. In addition, SNX10 is involved in intracellular vesicular trafficking and plays an important role in maintaining bone homeostasis [5]. To date, 16 variants in *SNX10* have been reported in 45 individuals with osteopetrosis, autosomal recessive 8, in the Human Gene Mutation Database (http://www.hgmd.cf.ac.uk/ac/index.php, last accessed April 20, 2022). Here, we describe a 4-year-old boy with a large homozygous deletion in the 5′-untranslated region (5′-UTR) of *SNX10* causing osteopetrosis autosomal recessive 8 with milder phenotypes.

## Methods

Written informed consent was obtained from participants before sample collection. Blood was drawn from the proband, and his parents in EDTA vacutainers. Genomic DNA was isolated by the phenol-chloroform method.

### Exome and genome sequencing and data analysis

Exome sequencing was performed using the Agilent SureSelect CREv2 Capture Kit and sequenced on the NovaSeq 6000 (Illumina Inc. USA).

Further, genome sequencing was performed with targeted coverage of 40× using NovaSeq 6000 (Illumina Inc. USA). Raw reads were aligned to the reference genome GRCh38. Delly was used to detect copy number variations and structural variations, and those variants involving known genes were further analyzed. A detailed protocol of data processing, variant calling, derivation of allele frequencies and allele states, and variant annotation was described in our previous study [6]. The UCSC browser was used to examine regulatory regions and to search for promoter/enhancer binding sites for the identified variants. The large deletion identified during genome sequencing was validated by GAP PCR with primers flanking the deleted region followed by Sanger sequencing.

### Reverse transcription PCR

Reverse transcription PCR (RT-PCR) was performed with RNA extracted from white blood cells using RiboPure™ RNA Purification Kit (Invitrogen), and cDNA was prepared using SuperScript^TM^ IV VILO^TM^ Master Mix (Invitrogen). Two different primer sets were used to perform RT-PCR (listed in Table 1). RT-PCR products were checked by 1% agarose gel electrophoresis.

**Table 1:** Primers for RT-PCR

Region	Primer type	Nucleotide sequence
Exon 1–7	Forward	TCCGGAACAACAGAAAGAGGA
	Reverse	TCTTCTTCGTACACAGGATGTT
Exon 6–7	Forward	AGACGTTTCCCTGAAGAAGATGAA
	Reverse	ACTGCTGTCATCACTACTGTGT

## Clinical report

**Fig. 1:**
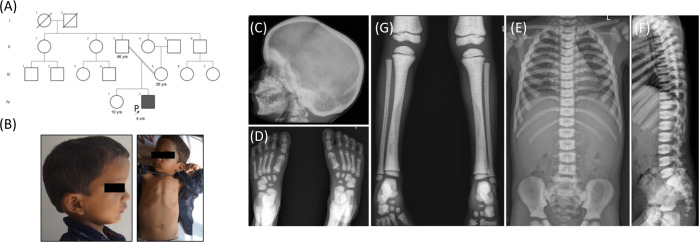
**A** Pedigree of the 4-year-old boy born to a second degree consanguineously married couple. **B** Radiographs showing that the proband has large skull, frontal bossing and pectus carinatum. **C**–**G** Radiographs at 4 years of age showing - (**C**) increased bone density at the outer cortex of the skull, (**D**, **E**) increase in bone mineral density at epiphyses and metaphyses. Irregular bone mineral density was noted at diaphysis, (**E**, **F**) increased thickness of vertebrae showing “sandwich” appearance of the vertebral plates

A 4-year-old male child of a second-degree consanguineous couple (Fig. 1A) was examined. He was born at term by normal vaginal delivery with a birth weight of 3.5 kg (−0.10 SD). At the age of 3 years, he was hospitalized for fever. At 4 years of age, he was 98 cm (−1 SD) tall, weighed 14.0 kg (−1.4 SD), and had a head circumference of 51 cm (+0.38 SD). Blood investigations revealed anaemia, thrombocytopenia and elevated lactate dehydrogenase (1047 U/L; reference value: 225–460 U/L). Physical examination revealed a large skull, frontal bossing and pectus carinatum (Fig. 1B). These observations were similar to those of affected individuals described in the previous reports [7, 8]. His hearing was normal, and the other developmental stages were also age-appropriate. We did not observe any visual impairment in him at this age. However, at the age of 5 years and 2 months, he developed a vision problem and bilateral optic atrophy was diagnosed. Hepatosplenomegaly and mesenteric lymphadenopathy were also noted. We found no neurologic abnormalities in the proband. Blood tests revealed a low haemoglobin level and decreased white blood cells and platelet counts. Further details of the biochemical investigations are given in Table 2.

**Table 2:** Biochemical investigations of the proband

Investigations	Value	Reference range
Haemoglobin	7.5 g/dL	11–14 g/dL
White blood cells	3.1 × 10^3^/ul	5–15 × 10^3^ul
Red blood cells	3.20 × 10^3^/ul	4–5.10 × 10^3^/ul
Platelets	128 × 10^3^/ul	200–490 × 10^3^/ul
Serum ferritin	47.7 ng/ml	13–400 ng/ml
Serum parathyroid hormone	23.8 pg/ml	18.4–80.1 pg/ml
Vitamin B12	725 pgm/ml	197–771 pgm/ml
Calcium	9.45 mg%	8.3–10.4 mg%
25-OH Vitamin D	17.01 ng/mL	20–32 ng/mL
Alkaline phosphatase	160 U/L	40–125 U/L
Creatinine	0.33 mg%	0.5–1.4 mg%

Complete skeletal examination revealed increased bone density at the outer cranial cortex, epiphyses, and metaphyses. A moderate increase in bone density and irregular transparency of the diaphysis were noted at the tibia. The proband was found to have an increased width of the ribs, and radiographs of the spine showed “sandwich vertebrae” and a bone-in-bone appearance (Fig. 1C–G).

**Fig. 2:**
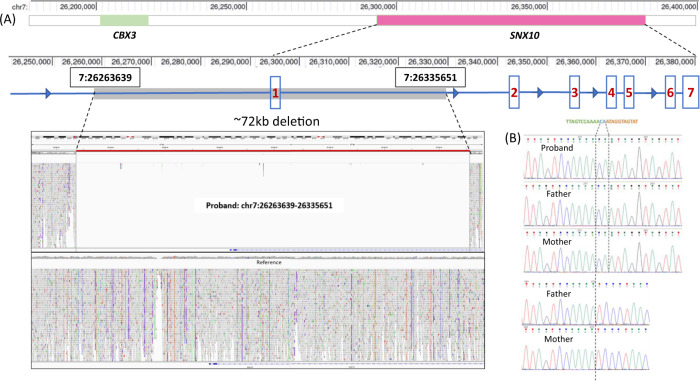
**A** Schematic representation showing ~72 kb indel variant upstream of *SNX10*. Screen capture of the BAM files analysis using the Integrative Genomics Viewer for the *SNX10* indel (g.26263639_26335651delinsCA) was found in the proband (**B**) Sanger chromatogram showing homozygous deletion and insertion of two nucleotides in the proband. parents are carriers of the identified variant

Exome sequencing revealed no significant disease-causing variants. Genome sequencing analysis using the in-house variant prioritization strategy revealed a large homozygous deletion of 72,012 bp, which was confirmed by manual analysis of the BAM file using Integrative Genomics Viewer. This deletion includes upstream region of *SNX10*, the noncoding exon 1, and a portion of intron 1 (Fig. 2A). GAP PCR and Sanger sequencing confirmed the deletion of 72,012 bp along with the insertion of two nucleotides (NC_000007.14:g.26263639_26335651delinsCA) in the proband (Fig. 2B). Both parents were carriers of the indel. This variant is not included in any of the control population databases.

**Fig. 3:**
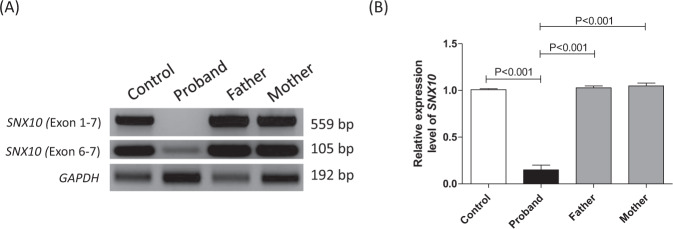
**A** Agarose gel electrophoretic separation of reverse transcriptase PCR (RT-PCR) amplicons encompassing exon 1 and 7 and exon 6 and 7 performed on cDNA samples of proband, carriers (parents) and control. Proband showed absence of *SNX10* transcript in the exon 1–7 primer set but lesser amount of transcript was observed in the exon 6–7 primer set; *GAPDH* represents control. **B** Densitometric quantification of the DNA bands obtained from RT-PCR with primers for exon 6 and 7 of *SNX10*

Further mRNA analysis using RT-PCR with the primer set for exon 1 and7 of *SNX10* showed the expected band size (559 bp) in the control, the absence of PCR product in the proband, while the parents showed normal DNA band size (559 bp) due to a normal allele in them. Another primer set including only exon 6 and 7 of *SNX10* showed a very weak band (105 bp) in the proband, indicating a lower amount of *SNX10* transcript compared to the control and healthy parents. RT-PCR for GAPDH was used for control normalization (Fig. 3).

## Discussion

We present a patient with autosomal recessive osteopetrosis 8. The clinical features of the proband are typical of the ARO phenotype such as increased bone density, large skull, bone-in-bone, thrombocytopenia, hepatosplenomegaly, loss of vision and “sandwich vertebrae”. We identified a deletion-insertion variant, NC_000007.14:g.26263639_26335651delinsCA in the homozygous state on chromosome 7 by genome sequencing. Exome sequencing failed to identify the large deletion because it encompasses a large noncoding region including the intergenic region upstream of *SNX10*, there showing that exome sequencing is not an ideal method for identifying large deletions in noncoding regions. Genome sequencing identified the exact breakpoints of the 72,012 bp long region encompassing the regulatory region of *SNX10*. The parents are healthy carriers of the same variant. Initially, we thought that due to the deletion of the 5′-UTR and exon 1 region of SNX10, there might be no SNX10 transcript, but a study by Ye et al., 2015 [9] showed severe osteopetrosis but no rickets in the osteoclast-specific SNX10 knockout. However, our patient showed a moderate phenotype without rickets. Further mRNA analysis revealed the presence of a lower amount of *SNX10* transcript. Therefore, we hypothesize that the decreased amount of *SNX10* transcript results in abnormal endosome sorting and vesicular transport to osteoclast bone lacunae.

Homozygous loss-of-function variants in *SNX10* leading to “osteoclast-rich” ARO were first described in Palestinian families [10]. It is estimated that *SNX10* is involved in 5% of all cases of ARO [7]. *SNX10* belongs to the family of sorting nexins that regulate endosome sorting and vesicular trafficking [11, 12]. SNX10 is also responsible for vesicular targeting of V-ATPase at the ruffled boundary. Loss-of-function variants in *SNX10* result in secondary impairment of V-ATPase and failure to acidify the resorption lacuna [13]. A homozygous ~70 kb deletion chr7:g.(26249558_26251671)-(26321193_26322492) in *SNX10* identified by 1 M array comparative genomic hybridization was previously reported with osteopetrosis [14]. Our ~72 kb deletion of the noncoding region partially overlaps with the reported ∼70 kb deletion. In our study, mRNA analysis was also performed by RT-PCR, which confirmed the deletion of the *SNX10* regulatory region, which had not been described previously. Non-coding region deletions involving regulatory elements have a significant impact on human development and health. Deleterious remote regulatory element mutations are well studied in the Van Buchem disease, a sclerosing bone dysplasia, and shown its effects on the transcriptional regulation of nearby genes.

Our study expands the molecular spectrum of *SNX10* mutations and identifies a novel mechanism of deletion of the upstream and untranslated region that is likely still underdiagnosed due to technical limitations. This study highlights the importance of genome sequencing for uniform coverage of noncoding regions, which helped us to identify the precise breakpoints of the identified deletion. In addition, our study fills the gap in studying the functional impact of a large deletion in *SNX10*.

## Web resources

1. PRIMER 3 v.4.1.0, http://primer3.ut.ee/
2. Ensembl, https://asia.ensembl.org/index.html
3. NCBI, https://www.ncbi.nlm.nih.gov/
4. OMIM, https://www.omim.org/
5. gnomAD, https://gnomad.broadinstitute.org/
6. HPO, https://hpo.jax.org/app/
7. ClinVar, https://www.ncbi.nlm.nih.gov/clinvar/
8. HGMD, http://www.hgmd.cf.ac.uk/ac/search.php
9. GATK, https://gatk.broadinstitute.org/
10. BWA, http://bio-bwa.sourceforge.net/
11. ANNOVAR, http://annovar.openbioinformatics.org/
12. DGV, https://clinicalgenome.org/tools
13. CROSSMAP, http://crossmap.sourceforge.net/
14. PanelApp, https://panelapp.genomicsengland.co.uk/
15. UCSC, https://genome.ucsc.edu/
16. Delly, https://github.com/dellytools/delly
